# Extensive migration of injected free liquid silicone for breast augmentation with related major complications

**DOI:** 10.1259/bjrcr.20150098

**Published:** 2015-05-15

**Authors:** J D Hilton, K Steinke

**Affiliations:** ^1^Department of Medical Imaging, Royal Brisbane and Women’s Hospital, Brisbane, QLD, Australia; ^2^Mayne Medical School, The University of Queensland, Brisbane, QLD, Australia

## Abstract

Free liquid silicone breast injections have been used for off-label breast augmentation since the 1960s. Shortly after the invention of this technique, multiple adverse effects became apparent and the technique became illegal in most countries. The procedure continues to be undertaken owing to its decreased cost compared with silicone prostheses. Complications from free silicone injections lead to complex management issues and health risks. This case demonstrates severe silicone migration, the extent of which has not previously been documented. In addition, the migration caused a serious life-threatening complication with subsequent complex management issues.

Breast augmentation surgery with injection of free liquid silicone has been performed from the early 1960s but was abandoned by most practitioners after a 1969 publication described multiple long-term adverse effects.^1^ The procedure consists of injecting medical or, in many cases, non-medical grade liquid silicone into the retromammary space, between the pectoralis major muscle and the fibroglandular breast tissue component. Despite multiple known associated complications, the procedure remains available in parts of Asia, Eastern Europe and South America largely owing to its low cost. This case outlines a patient who underwent an elective mastopexy followed by breast augmentation with free liquid silicone. Silicone migration has previously been described; however, to our knowledge, a migration of this extent has not been recorded.^2^ In addition, a severe life-threatening complication owing to silicone migration, as depicted in this case, has not been described elsewhere.

## Case report

**Figure 1.**
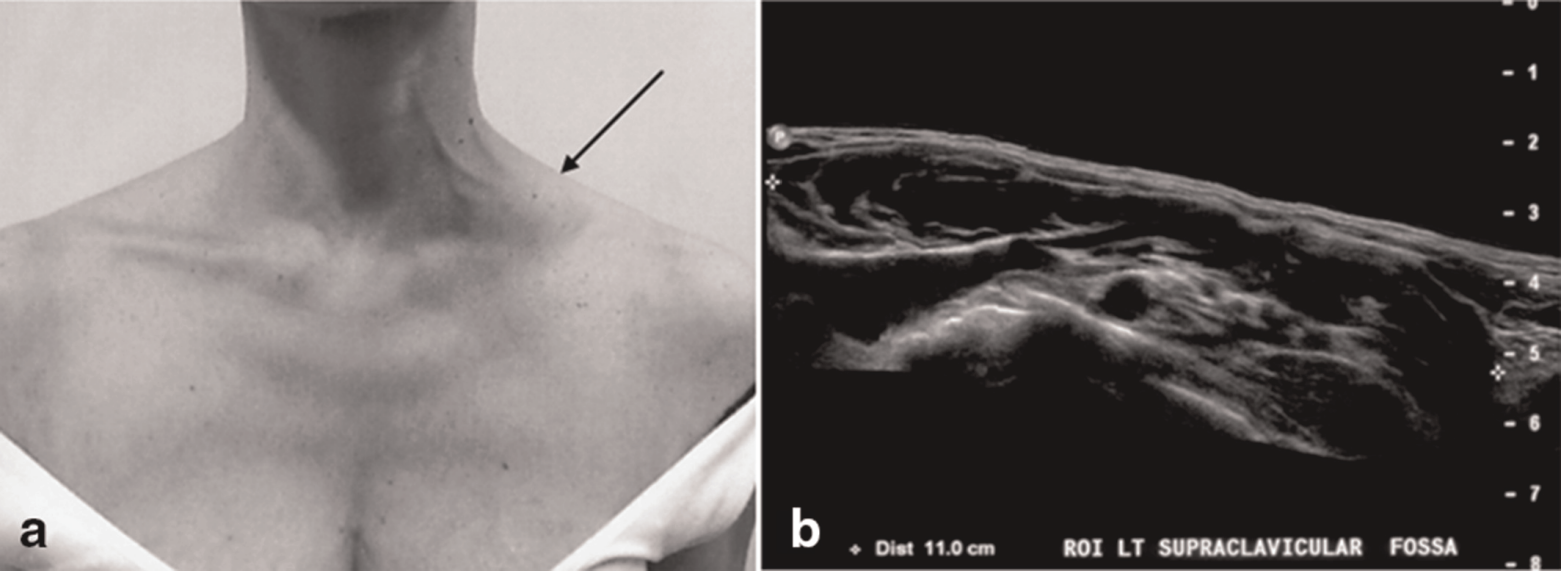
(a) Supraclavicular oblong mass (arrow) as noticed by patient at the time of presentation. (b) Hypoechoic, approximately 11-cm oblong mass in the left supraclavicular fossa demonstrated on 12-MHz linear probe ultrasound.

A 46-year-old female presented to her general practitioner with a painless left supraclavicular mass (Figure 1a). No further history was provided at the time. Initial imaging was performed with both ultrasound (Figure 1b) and CT. The CT was performed as a non-contrast scan, as per the patient’s request. The lack of intravenous contrast made interpretation of the mass difficult and the study was of little help forming a differential diagnosis, which from the ultrasound was lymphangioma or cystic hygroma.

The patient was referred to interventional radiology for further investigation and possible treatment of the presumed lymphangioma.

On targeted investigation, she reported having had an elective mastopexy in Russia 3 years prior. Postoperatively, the patient was not satisfied with the result and desired a fuller appearance. The surgeon suggested free liquid silicone injection, as he claimed to have had good aesthetic outcomes from this technique in the past. As the procedure was carried out in Russia, no details were available as to the volume and grade of silicone injected. The patient reports having had an aesthetically pleasing result after the injections. The patient’s history included recent domestic violence, but breast trauma was denied. No other significant history was provided. Upon examination, in addition to the soft oblong left supraclavicular mass, the left breast was noted to be slightly smaller than the right one. No other masses were palpated.

**Figure 2.**
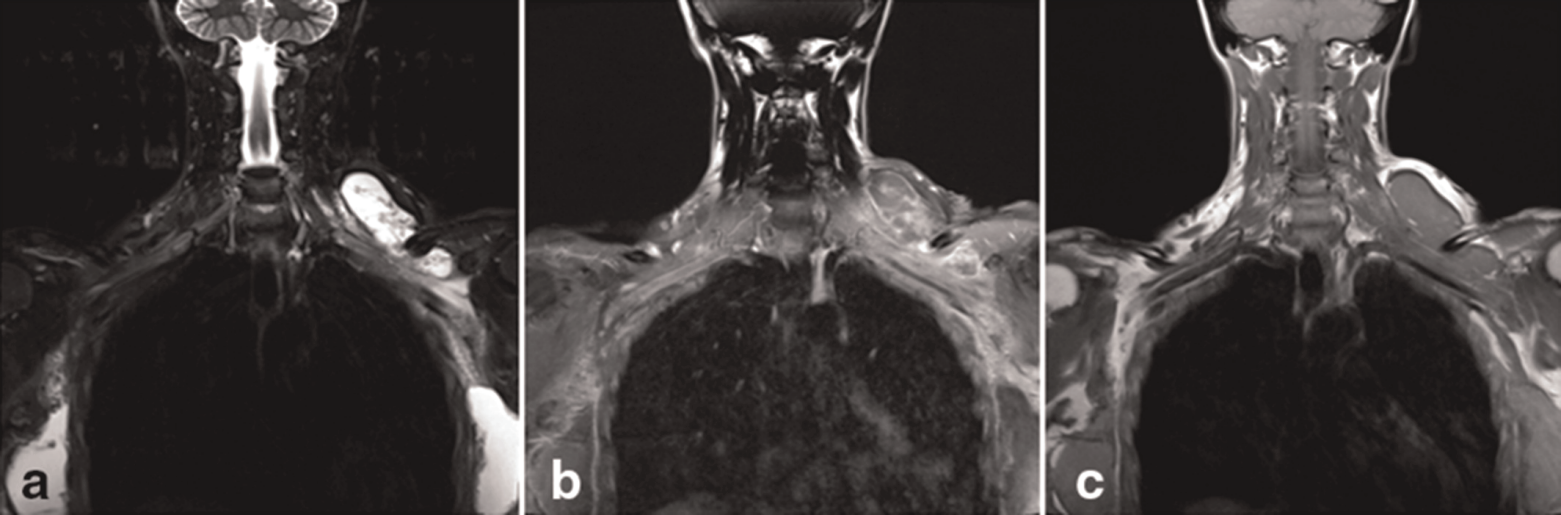
MRI on presentation, coronal reformats showing a (a) short tau inversion-recovery (STIR) hyperintense mass in the left supraclavicular fossa. Although no silicone-excited sequences were performed, the material was of the same intensity as the breast material and a diagnosis of silicone migration was made. (b) Pre-contrast *T*_1_ weighted TSE scan in which the isointense mass is evident, however, not clearly distinguishable from surrounding tissue. (c) Post-contrast *T*_1_ weighted TSE fat-saturated image that allows the mass and further components along the chest wall to be visualized. TSE, turbo spin echo.

An MRI was performed (3T Siemens Trio Tim magnet; Siemens Healthcare, Erlangen, Germany) (Figure 2) to further define the mass and its vascularity before treatment. Pre- and post-contrast *T*_1_, *T*_2_ and short tau inversion-recovery (STIR) weighted sequences were performed. Imaging showed a well-defined, *T*_2_ and STIR high signal, complex cystic lesion extending from the lower anterior left neck posteriorly into the supraclavicular fossa (Figure 2a). An asymmetry of the injected material into both breasts was also noted, with silicone migrating around the lateral border of the left pectoralis major muscle. A diagnosis of liquid silicone migration was made.

At this time, bilateral mastectomy was advised with cosmetic reconstructions. The patient declined this surgery.

**Figure 3.**
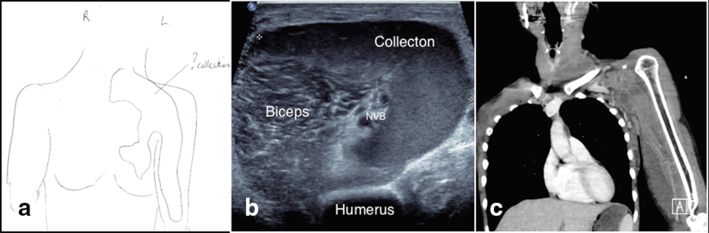
Images from the second presentation. (a) Schematic drawing from sonographer depicting the extent of the collection through the anterior chest wall, axilla and arm. (b) Ultrasound scan, transverse plane, shows large, heterogenous collection within the proximal medial portion of the left arm. It encases the NVB and displaces the biceps muscle. It is not characteristic for silicone, as it does not produce the typical "snow storm" artefact. (c) Post-contrast CT coronal reformat confirms a large wall-enhancing heterogenous collection extending between the neck and the swollen arm, as demonstrated in the previous ultrasound. NVB, neurovascular bundle.

Nearly 1 year later, the patient represented, acutely unwell, septic, with a swollen, tense left biceps region and left upper limb cellulitis. Blood tests confirmed a staphylococcus bacteraemia with a white cell count of 18.6 × 10^9^ l^−1^ (4–11), neutrophils of 16.9  × 10^9^ l^−1^ (2–9) and a C-reactive protein of 358  mg l^−1^ ( <5), normal ranges are given in brackets. An ultrasound scan was performed to exclude abscess formation, and it demonstrated a complex, heterogenous collection with nodular internal echogenic material, extending from the left supraclavicular fossa into the left chest wall as well as along the proximal, medial part of the left arm (Figure 3a,b). Although the history of silicone migration was known, the volume of the collection visualiszed on this presentation appeared to outweigh the presumed volume of injected silicone material.

After consenting to intravenous contrast administration, she progressed to have a CT of the neck and left upper limb with split bolus intravenous contrast (Figure 3c). This showed extensive hypodense material within the distribution of the previously identified silicone migration site with a new distribution of hypodense material in the left arm, correlating well with the ultrasonographical findings. A slightly enhancing rim surrounding the arm collection suggested superimposed inflammation or infection.

**Figure 4.**
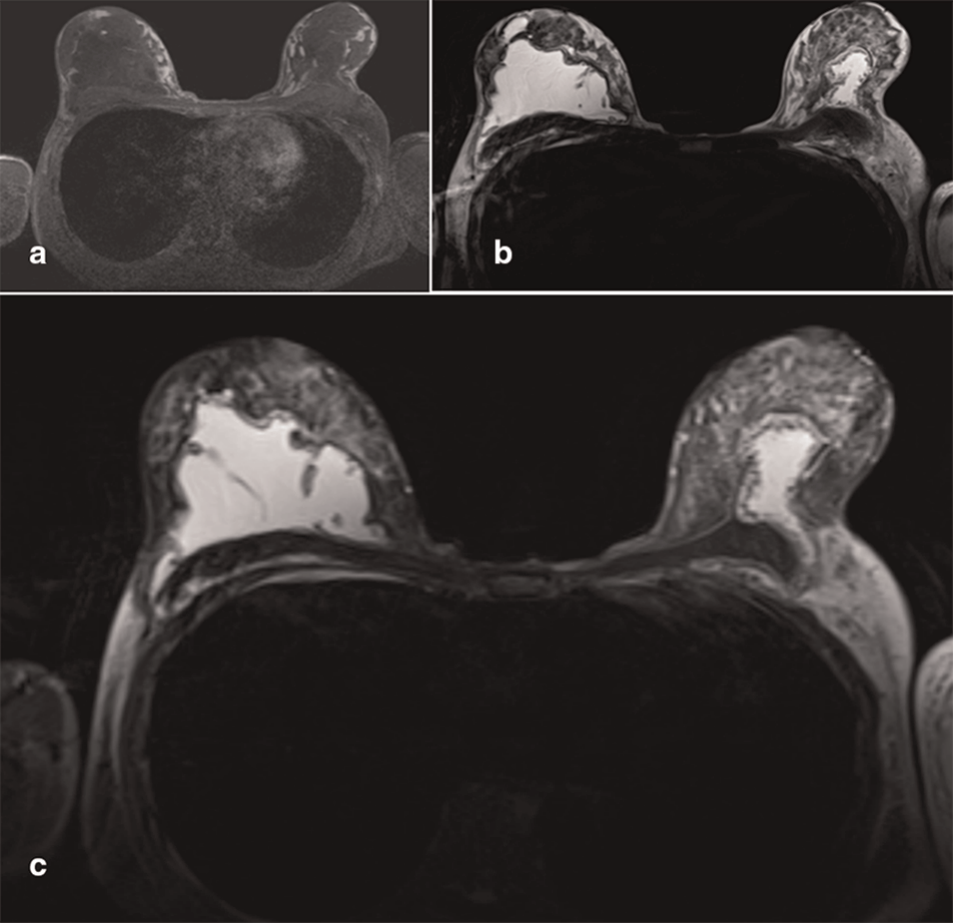
Axial non-contrast enhanced MR breast images. (a) *T*_1_ non-fat-saturated image showing isointense tissue in both breasts; note asymmetry with the right breast larger than the left and left lateral pectoral/anterolateral chest wall fullness. (b) *T*_2_ silicone-excited image shows high signal material in predominantly retroglandular location, also wrapping around the lateral edge of the right major pectoral muscle; slightly hyperintense tissue in the left chest wall adjacent to the pectoral muscle, different signal to intramammary tissue. (c) Turbo inversion recovery magnitude water-saturated silicone-excited scan allows to discriminate between silicone and other fluid; strongly hyperintense material represents silicone. The paucity of silicone within the left breast and the migration around the left pectoral muscle is noteworthy.

**Figure 5.**
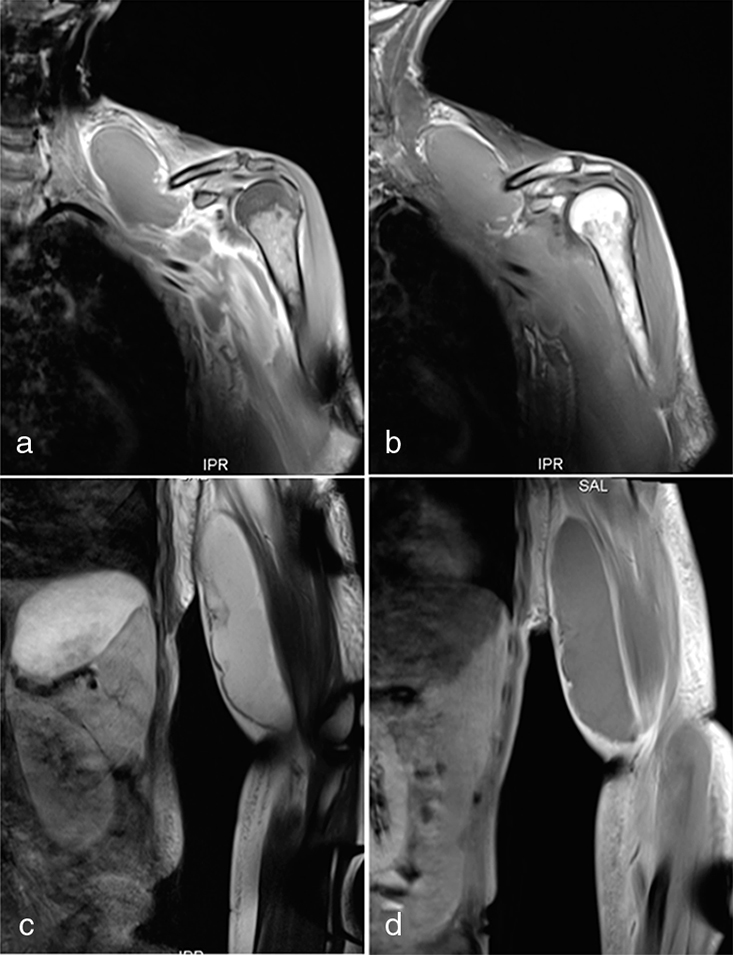
MRI coronal reformats from the neck and left arm demonstrating a large supraclavicular mass extending into the arm. (a) *T*_1_ weighted TSE pre-intravenous-contrast, (b) *T*_1_ weighted TSE sequence post-gadolinium contrast with fat saturation. The post-contrast study clearly shows the known silicone collection in the supraclavicular fossa tracking into the axilla and proximal arm and defines the planes between the collection and muscles. (c) *T*_2_ fat-saturated TSE scan and (d) post-contrast *T*_1_ TSE fat-saturated scan. TSE, turbo spin echo.

The following day, an MRI was performed to delineate between the silicone and the infective process in the arm (Figure 4). The volume of silicone within the left breast had decreased significantly from the previous MRI, and silicone was identified throughout the supraclavicular fossa migrating into the biceps compartment of the left arm (Figure 5). This migrated liquid silicone was now compressing the adjacent lymphatic drainage as well as the veins. Surrounding the migrated silicone were several multiloculated mildly rim-enhancing collections, not following the signal of silicone.

The finding of suspected abscess collections was relayed to the treating surgical team and a large volume of pus (totalling approximately 600 ml) was drained. A CT sinogram was performed that, as expected, demonstrated a fluid pocket extending from the medial aspect of the left arm to the left axilla and supraclavicular fossa. The patient was placed on intravenous cephalexin followed by a long course of oral flucloxacillin. Following this major infective process in her arm, the patient agreed to bilateral mastectomy with reconstruction.

## Discussion

Use of liquid silicone injections for cosmesis continues to be a widely used technique, although the Food and Drug Administration only approves medical grade silicone for treatment of retinal detachment. Use for augmentation is off-label and silicone used for large volume augmentation is regularly non-medical grade. It has previously been used for treatment of acne scarring on the face, facial, breast and buttock augmentation and treatment of diabetic foot ulcers. Originally, in the 1960s, silicone was thought to be inert, and at this time between 20,000 and 40,000 patients in the USA alone were injected for breast augmentation with volumes of up to 2000 cc in each breast. As complications became apparent, the formula of silicone was altered, including mixing it with olive oil, in an attempt to induce fibroplasia. This formula (Sakurai formula) was used in over 100,000 patients, with no change in adverse effects.^3^ Two greatly different methods of silicone injection have been described for breast augmentation, one involving medical grade silicone with microdroplet technique and the other with large volumes of industrial grade silicone injected by practitioners who can be either unlicensed or unskilled.^3^ Both techniques are demonstrated to cause similar adverse effects and have since been declared illegal in most countries. The procedure continues to be in demand as the cost is significantly less than contained silicone implants (complications of which will not be discussed).

Liquid silicone breast injections have a variety of known, well-documented side effects, including mastodynia, granuloma formation, skin discolouration, skin irregularities and mastitis. In addition, a more serious complication of pneumonitis secondary to silicone liquid-induced emboli is well documented.^4^ Pulmonary complications usually occur within few days of free silicone injection.^4^ Silicone embolism has been likened to fat embolism regarding the pathomechanism and clinical presentation.^5^ Multiple deaths have occurred because of liquid silicone injections, all of which have been from respiratory failure, in the form of silicone emboli. Each death was associated with a large volume of injected contrast and led to criminal charges in the USA.^3^

In a case series of 28 patients, the average time between treatment and complication was 9 years, the earliest occurred within 1 year from injection, while the latest was recorded at 20 years.^6^ Despite the most common complaint being mastodynia, the more clinically challenging complaint is from granuloma formation within the breast and, at times, along the chest wall and in the supraclavicular region.^5^ Granulomata are clinically indistinguishable from breast cancers, as both form hardened, irregular masses. Furthermore, the fact that the silicone obscures the breast parenchyma renders mammographies virtually non-diagnostic^7^; the accessibility with ultrasound is also impaired owing to posterior shadowing characteristics termed “snow storm” appearance.

MRI is a superior diagnostic imaging modality in cases of free liquid breast augmentation. Using a combination of fat suppression, water suppression, *T*_1_, *T*_2_ and silicone weighting, one is able to distinguish between a wide variety of materials used to augment the breasts through direct injection, such as free silicone, paraffin, saline or autologous fat.^8^ In addition, MRI is able to differentiate between reactive silicone granulomatous tissue and breast cancer, even in cases where the two entities are in close proximity.^9^ This is an obvious advantage over ultrasound or CT scan.

The surgeons involved in this case describe removal of free liquid silicone as a challenging procedure owing to the adhesive nature of silicone to the surrounding tissue. In addition, preservation of tissue for reconstructive purposes can be difficult owing to extensive infiltration through the injected area.

## Learning points

1. Migration of liquid silicone beyond the axilla constitutes a potentially major complication that requires consideration of surgical intervention.
2. Comprehensive imaging is mandatory and is best achieved with dedicated MRI imaging, which is superior to alternative imaging modalities for assessing nature, delineation and extent of the migrated silicone and associated complications.
